# A Role for the Non-Receptor Tyrosine Kinase Abl2/Arg in Experimental Neuroinflammation

**DOI:** 10.1007/s11481-018-9783-8

**Published:** 2018-03-17

**Authors:** Freja Aksel Jacobsen, Alexander N. Scherer, Jeppe Mouritsen, Hera Bragadóttir, B. Thomas Bäckström, Samra Sardar, Dan Holmberg, Anthony J. Koleske, Åsa Andersson

**Affiliations:** 1 Department of Drug Design and Pharmacology, Faculty of Health and Medical Sciences, University of Copenhagen, Copenhagen, Denmark; 2 Present Address: Novo Nordisk A/S, Bagsværd, Denmark; 3 Department of Cell Biology, Yale University School of Medicine, New Haven, CT USA; 4 Present Address: Novozymes A/S, Bagsværd, Denmark; 5 Present Address: Xellia Pharmaceuticals A/S, Copenhagen, Denmark; 6 Novo Nordisk A/S, Måløv, Denmark; 7 Present Address: BTB Pharma, Malmö, Sweden; 8 Autoimmunity section, CRC, Lund University, Malmö, Sweden; 9 Department of Molecular Biophysics and Biochemistry, Yale University School of Medicine, New Haven, CT USA; 10 Rydberg laboratories for Applied Science, Academy of Business, Technology and Science, University of Halmstad, Halmstad, Sweden

**Keywords:** Abl kinase, Arg, *Eae27*, Experimental autoimmune encephalomyelitis, Imatinib

## Abstract

Multiple sclerosis is a neuroinflammatory degenerative disease, caused by activated immune cells infiltrating the CNS. The disease etiology involves both genetic and environmental factors. The mouse genetic locus, *Eae27*, linked to disease development in the experimental autoimmune encephalomyelitis (EAE) model for multiple sclerosis, was studied in order to identify contributing disease susceptibility factors and potential drug targets for multiple sclerosis. Studies of an *Eae27* congenic mouse strain, revealed that genetic variation within *Eae27* influences EAE development. The *Abl2* gene, encoding the non-receptor tyrosine kinase Arg, is located in the 4,1 megabase pair long *Eae27* region. The Arg protein plays an important role in cellular regulation and is, in addition, involved in signaling through the B- and T-cell receptors, important for the autoimmune response. The presence of a single nucleotide polymorphism causing an amino acid change in a near actin-interacting domain of Arg, in addition to altered lymphocyte activation in the congenic mice upon immunization with myelin antigen, makes *Abl2/Arg* a candidate gene for EAE. Here we demonstrate that the non-synonymous SNP does not change Arg’s binding affinity for F-actin but suggest a role for Abl kinases in CNS inflammation pathogenesis by showing that pharmacological inhibition of Abl kinases ameliorates EAE, but not experimental arthritis.

## Introduction

The complexity of autoimmune diseases, involving both genetic and environmental factors, makes it difficult to reveal mechanisms responsible for initiating an immune response against self-structures. Multiple sclerosis (MS) is a disabling neurodegenerative disease caused by an immune response against the myelin sheath surrounding the neuronal axon. The disease affects 2.3 million people worldwide with a higher disease incidence in females (Harbo et al., 2013). Genetic variations within the major histocompatibility complex (MHC) have been linked to MS (Rioux et al., 2009; Patsopoulos et al., 2013). Furthermore, genome-wide association studies have identified non-MHC-genes as disease causing candidate genes (International Multiple Sclerosis Genetics C et al., 2007; ANZgene, 2009; Baranzini et al., 2009; International Multiple Sclerosis Genetics C, 2013).

Genetic studies in animal models allow a homogeneous and well-controlled environment, where variation within test groups can be minimized. One strategy to study genetic factors linked to disease susceptibility is breeding of congenic mouse strains followed by studies of disease development in models resembling human inflammatory disease. This strategy enables studies of specific genetic loci, and the impact of therein located genes, and natural allelic variation, on disease development (Rogner and Avner, 2003). The RIIIS/J mouse strain is, in contrast to the B10.RIII strain, resistant to experimental autoimmune encephalomyelitis (EAE), the animal model for MS, when immunized with myelin basic protein peptide 89–101 (MBP_89–101_) (Jansson et al., 1991). The B10.RIII and RIIIS/J mouse strains both express the murine H-2^r^ MHC haplotype, concluding that non-MHC genes are responsible for the difference in disease phenotype observed in the two strains (Sundvall et al., 1995; Karlsson et al., 2003; Lindvall et al., 2011).

Linkage analyses have revealed novel Quantitative Trait Loci (QTLs) for EAE susceptibility in mice. The QTL *Eae27*, located on mouse chromosome 1, has been linked to EAE susceptibility upon disease induction with MBP_89–101_ (Karlsson et al., 2003). The gene encoding the Abl2/Arg non-receptor tyrosine kinase is located within *Eae27* (Kruh et al., 1986). Together with the Abl1/c-Abl kinase, Abl2/Arg makes up the family of Abl tyrosine kinases in vertebrates and the two kinases have both overlapping and distinct functions in the cell (Bradley and Koleske, 2009; Colicelli, 2010). Abl kinases have been found to play essential roles for the downstream signaling of the T- and B-cell receptors (Zipfel et al., 2000; Bianchi et al., 2002; Zipfel et al., 2004; Gu et al., 2007). The kinases share significant sequence conservation in their N-terminal halves, comprising tandem Src homology (SH) 3, SH2, and kinase domains, while the two proteins diverge significantly in their C-terminal halves (Colicelli, 2010). The Arg C-terminal contains two F-actin binding domains and a microtubule-binding domain (Wang et al., 2001; Miller et al., 2004) and is a key regulator of actin cytoskeletal rearrangements (Hernandez et al., 2004; Miller et al., 2004; Miller et al., 2010; Wang et al., 2010). Establishment of an immune response involves morphological changes of immune cells and migration, adherence, invasion, in addition to cell-cell interactions through immunological synapse formation, are cellular processes relying on reorganization and modulation of the cellular actin cytoskeleton (Burkhardt et al., 2008; Huang et al., 2008). As a key node in immune cell signaling, as well as a direct regulator of actin dynamics and microtubule distribution in cells (Wang et al., 2001; Miller et al., 2004), Arg could play a central role for the cellular mechanisms leading to a self-directed immune response observed in EAE and MS.

Here we report that a congenic mouse strain, BR.RIIIS/J-*Eae27*, which harbors a 4,1 Megabase pair (Mbp) polymorphic region, including the *Abl2/Arg* gene, develops less progressive EAE and shows altered T- and B-cell in vitro phenotypes, compared to littermate controls. We show that treatment with an Abl kinase inhibitor ameliorates EAE progression, indicating a role for Abl kinases in EAE pathogenesis. Moreover, we report that a non-synonymous single-nucleotide polymorphism (SNP) in the *Abl2* gene, which may influence the function of the protein and putatively EAE development, is not important for the actin-binding capacity of Arg.

## Methods

### Animals

B10.RIII mice were originally provided by Dr. J. Klein (Tübingen, Germany). The RIIIS/J strain originated from the Jackson Laboratory (Bar Harbor, ME, US). Congenic BR.RIIIS/J-*Eae27* and B6.RIIIS/J-*Eae27* mice were bred by introduction of *Eae27* from the EAE resistant RIIIS/J donor strain to the EAE susceptible B10.RIII strain through backcrossing for a minimum of seven generations with the marker-associated speed congenic strategy (Markel et al., 1997; Wakeland et al., 1997). Genotyping using microsatellites allowed selection for the *Eae27* locus inherited from the RIIIS/J strain. Microsatellite markers D1Mit14 and D1Mit33 (www.informatics.jax.org) were used for PCR on DNA from ear tissue biopsies as previously described (Karlsson et al., 2003). Genotypes were determined by analyzing PCR products on a Megabace 1000 (Amersham Pharmacia Biotech, Amersham, UK) or on a 3% agarose gel.

Congenic mice, homozygous for *Eae27* introduced from the RIIIS/J donor strain to the B10.RIII (BR) genetic background, are referred to as BR.RIIIS/J-*Eae27*. Congenic littermates, homozygous for *Eae27* inherited from the background strain, are referred to as B10.RIII. Breeding and housing of congenic mice used in the herein presented study, took place in the animal facility at the Department of Drug Design and Pharmacology, Faculty of Health and Medical Sciences, University of Copenhagen, Denmark.

### Induction and Evaluation of EAE

Mice were immunized id at the base of the tail with 150 μg MBP_89–101_ (Schafer, Denmark) in PBS emulsified 1:1 in Freund’s incomplete adjuvant (IFA) (Sigma-Aldrich, St. Louis, MO) complemented with 100 μg heat-inactivated mycobacteria tuberculosis H37Ra (DIFCO laboratories, Detroit, MI). 400 ng Pertussis toxin from Bordetella Pertussis (Sigma-Aldrich, St. Louis, MO) in PBS was administered ip at the day of immunization and 2 days post immunization. Disease progression was monitored by daily scoring: (0) No clinical symptoms; (1) loss of tail tonus; (2) mild paresis in one or both hind legs; (3) moderate paresis in one or both hind legs; (4) severe paresis in one or both hind legs; (5) paresis in one or both hind legs and any significant paresis in front leg; (6) moribund or diseased. According to an approved study protocol defining humane endpoints, mice receiving scores above 4, or losing more than 20% of their initial body weight, were euthanized. Animal Experiments Inspectorate permission number: 2010/561–1920.

### Induction and Evaluation of CIA

For induction of collagen-induced arthritis (CIA), B10.RIII mice were immunized id at the base of the tail with 100 μg type II collagen from bovine nasal septum (Sigma-Aldrich, St. Louis, MO) in 0.05 M acetic acid emulsified 1:1 in complete Freund’s adjuvant (CFA). A boost with half the concentration of collagen (50 μg) was administered 35 days post first immunization. Scoring of clinical symptoms monitored disease progression. Each inflamed toe (first phalanx excluded), and each inflamed metacarpal, carpal, metatarsal and tarsal, respectively, was assigned 1 point. Mice receiving a score above 10 were euthanized according to predefined and Animal Experiments Expectorate approved humane endpoints. Animal Experiments Inspectorate permission number: 2010/561–1920.

### Bioinformatics and SNP Analysis

A complete literature search on genes within *Eae27* was performed. Key words like EAE, MS, autoimmunity, chronic inflammation, and immune cell signaling were used to identify genes likely to play a role in immune regulation. SNP analysis using Ensembl’s and Mouse Genomic Informatics’ SNP databases, combined with next generation sequencing (NGS) data (performed at the National High-throughput DNA sequencing center, Copenhagen University) revealed SNPs within *Eae27*, when comparing the genomes of the two parental strains. Sanger sequencing (Eurofins, Germany), was carried out on DNA from each parental strain to confirm identified SNPs in the coding regions of the *Abl2* gene.

### Pharmacological Studies

Imatinib mesylate (100 mg/kg body mass), kindly donated by Novartis, Basel, Switzerland, was administered to B10.RIII mice orally by gavage twice daily with a 12 h interval to maintain a therapeutic dose. The drug was solubilized in water prior to administration, and mice received the drug 1 day prior to EAE or CIA immunization and continuously throughout the entire study. A control group received placebo (water) on an identical treatment regimen.

### Preparation of Spleen Cells and Proliferation Assay

Single-cell suspension was prepared from mouse spleen in complete cell culture medium (Dulbecco’s modified eagle’s medium + GlutaMAX-I, 1% fetal bovine serum, 1 mM Hepes, 5 μM 2-mercapto-ethanol, and 50,000 U penicillin/50 mg streptomycin) (Invitrogen, Thermo Fisher Scientific, Waltham, MA). Red blood cells were lysed in BD Pharm Lysing Buffer (BD Biosciences, San Jose, CA).

Splenic lymphocytes from naive or MBP_89–101_ immunized BR.RIIIS/J-*Eae27* and B10.RIII mice were collected 10 days post immunization. Single-cell suspensions (2 × 10^5^ cells/well) were stimulated in vitro with purified anti-mouse CD3 (BD Biosciences, San Jose, CA) and anti-mouse CD28 (eBioscience, San Diego, CA); Concanavalin A (ConA) (Sigma-Aldrich, St. Louis, MO); lipopolysaccharide (LPS) (Sigma-Aldrich, St. Louis, MO); or F(ab’)2 Fragment Goat Anti-Mouse IgM (Jackson Immuno Research, West Grove, PA) for 48 h. Cells were subsequently pulsed with 1 μCi 3H-thymidine (Perkin Elmer, Waltham, MA) per well for 16–18 h, and ^3^H-thymidine incorporation monitored on a TopCount NXT (Perkin Elmer, Waltham, MA). Stimulation index (SI) was calculated as the ratio between counts per minute (cpm) for stimulated and unstimulated cells. For inhibition studies, sterile filtrated imatinib in PBS was added to the cell suspension prior to incubation.

### Western Blotting

Upon cardiac perfusion, mouse brain and spleen were isolated and snap frozen in liquid nitrogen. Tissue was homogenized in lysis buffer (50 mM Tris, pH 8, 150 mM NaCl, 0.5% sodium deocholate, 1% Triton X-100, 0.1% SDS) to which phosphatase and protease inhibitors had been added. The total protein concentration was determined by Bradford assay, and samples with a total protein conc. of 1 μg/μl were separated on an 8% SDS-PAGE gel and bands detected by western blotting using monoclonal rabbit anti-mouse Arg, monoclonal rabbit mouse anti-beta-actin (loading control), and polyclonal goat anti-rabbit IgG-HRP used for detection.

### Actin Co-Sedimentation Assays

Actin was purified from chicken skeletal muscle (Spudich and Watt, 1971). Arg and Arg V1030 M were expressed via baculoviral vectors in Hi5 cells and purified as previously described (Wang et al., 2001). Cosedimentation assays were performed as previously described with a final buffer concentration of 40 mM Hepes, pH 7.25, 150 mM KCl, 2 mM MgCl_2_, 0.01% NP40, and 4.5% glycerol. Supernatant and pellet were separated on SDS PAGE gel, stained and quantified using Coomassie R250 and densitometry.

### Statistics

In vivo and in vitro data were calculated with Mann-Whitney U test, except for CIA data, where statistical analysis was performed using the log-rank test. Disease incidence was calculated with chi-square test.

## Results

### Severity of EAE Is Influenced by Genetic Variation in the *Eae27* Locus

**Fig. 1:**
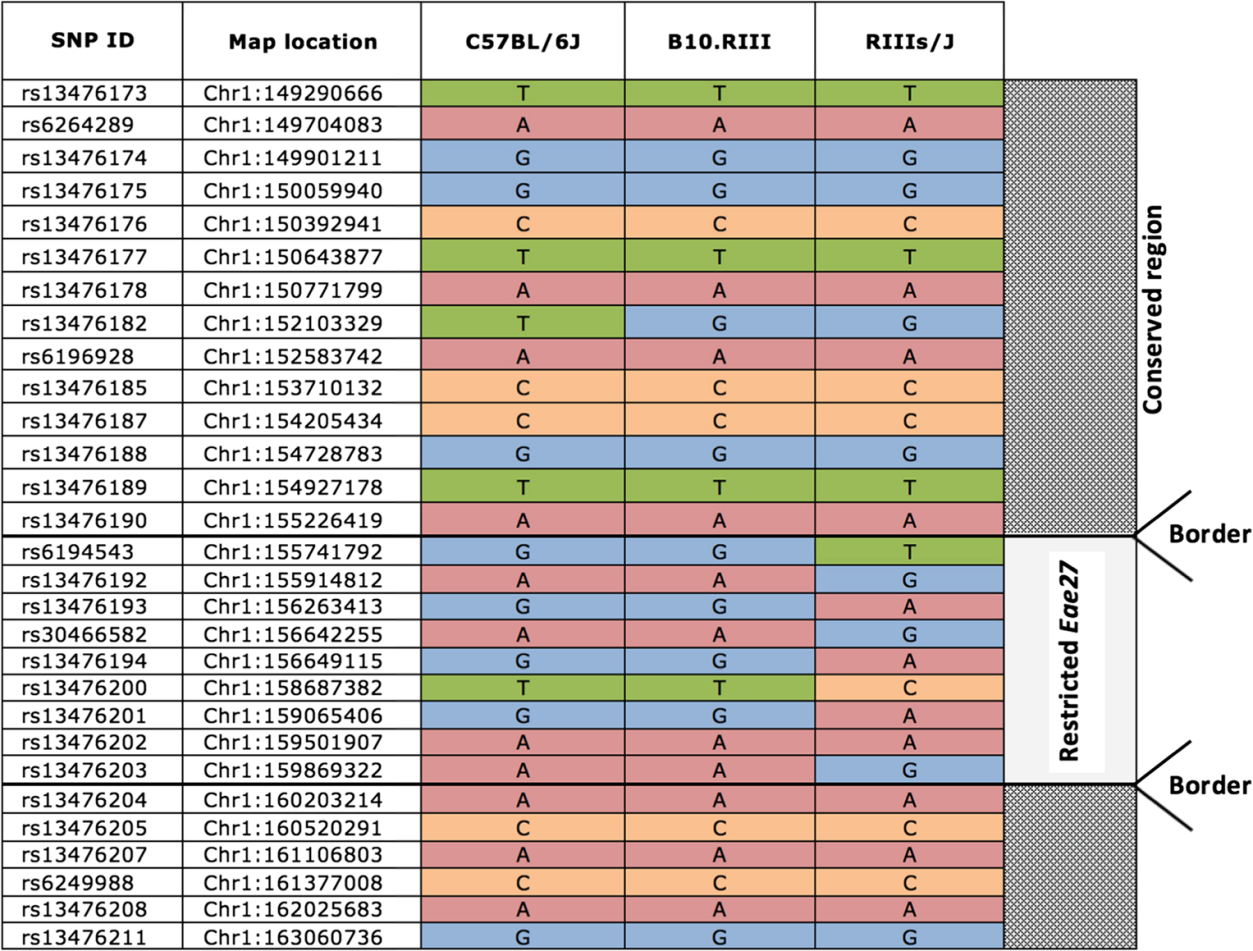
***Eae27*** **SNP analysis**. SNP analysis revealed known SNPs within the *Eae27* locus comparing the C57Bl/6 and RIIIS/J mouse strains. Identified SNPs were subsequently compared to next generation sequencing data for the B10.RIII strain and led to the identification of the restricted polymorphic *Eae27*, flanked by highly conserved regions

In a genetic linkage study, *Eae27* was linked to incidence of remitting EAE in female mice (Karlsson et al., 2003). In order to study the effect of this locus for EAE development, a congenic mouse strain was bred (BR.RIIIS/J-*Eae27*). The congenic fragment was estimated to include 15 Mbp between the genetic markers D1Mit449 and D1Mit143 (www.informatics.jax.org). Comparison of SNPs between the two parental mouse strains revealed conserved and polymorphic regions and reduced the locus size to approximately 4.1 Mbps (Fig. 1).

**Fig. 2:**
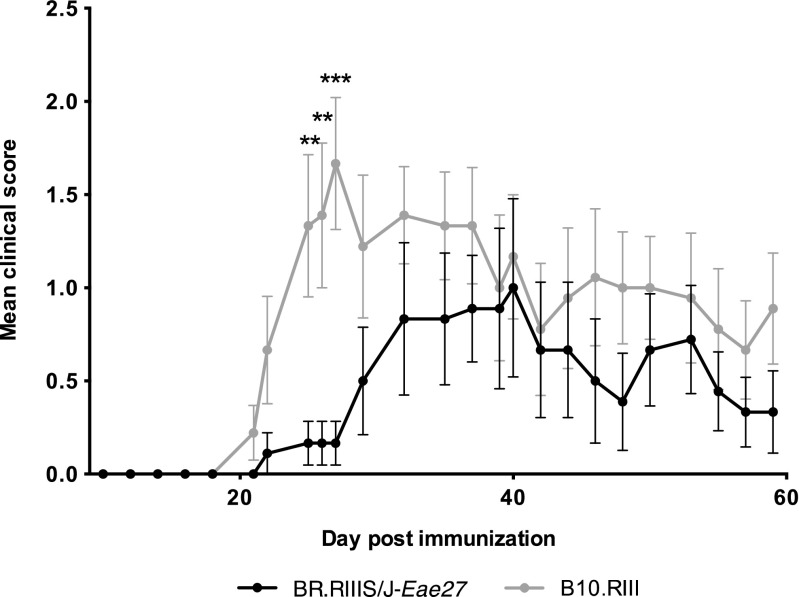
**EAE development in BR.RIII-*Eae27*** **congenic mice**. Disease progression in mice homozygous for *Eae27*, inherited from the EAE susceptible B10.RIII background strain (B10.RIII; grey line) and the EAE resistant RIIIS/J donor strain (BR.RIII-*Eae27*; black line). Data are presented as mean clinical score ± SEM

BR.RIIIS/J-*Eae27* mice and littermate controls were immunized for EAE and the disease progression was monitored by clinical scoring (Fig. 2). The result revealed significantly lower severity of disease in BR.RIIIS/J-*Eae27* mice (*p* = 0.0009, at day 27 post immunization). Reduced incidence and area under curve (AUC), defined as the sum of scores over the period, was observed in BR.RIIIS/J-*Eae27* mice with one or two *Eae27* RIIIS/J alleles, compared to B10.RIII littermates (*p* = 0.012) (Table 1). Similar results were obtained in an additional EAE study with the same experimental set-up (data not shown). This shows that genetic variation within the restricted *Eae27* locus influences EAE susceptibility and progression.

**Table 1:** Disease phenotypes in BR.RIIIS/J-*Eae27* congenic mice immunized for EAE

Mouse strain	n	Incidence	Day of onset^a^	Max Score^c^	AUC^d^
			Mean ± SEM^b^	Mean ± SEM	Mean ± SEM
BR.RIIIS/J-*Eae27*_*a/a*^e^	9	6/9 (67%)	30 ± 2,3	1,6 ± 0,5	11,7 ± 4,2
BR.RIIIS/J-*Eae27_a/b*	20	12/20 (60%)	27 ± 2,4	1,4 ± 0,3	11,5 ± 3,5*
BR.RIIIS/J-*Eae27_b/b*	9	9/9 (100%)	26 ± 2,7	2,2 ± 0,3	25,4 ± 6,5

^a^Day of onset is the day when a mouse receives score 1 or higher

^b^Standard Error of the Mean

^c^The mean of max scores from the mice in each group

^d^Area under curve is the sum of all scores

^e^Genotype in the *Eae27* locus. All mice studied were littermates

*p < 0,05; significantly different from the BR.RIIIS/J-*Eae27_b/b* mouse strain. Statistics was calculated with Mann-Whitney U test

### Increased Activity in Lymphocytes from Immunized *Eae27* Congenic Mice

**Fig. 3:**
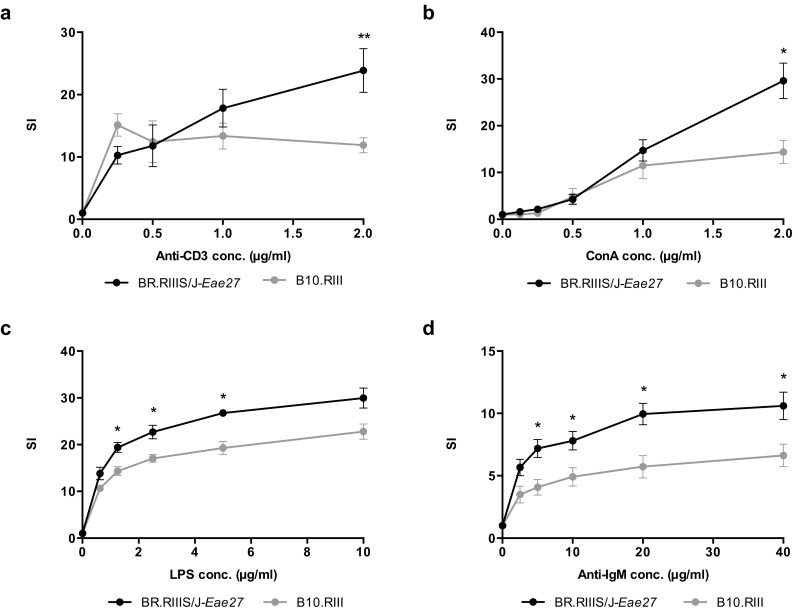
**Proliferation of spleen cells from mice immunized with MBP**_**89–101**_ **upon activation in vitro**. ^3^H-thymidine incorporation in in vitro stimulated splenic lymphocytes from MBP_89–101_-immunized BR.RIIIS/J-*Eae27* and B10.RIII mice. The graphs show T-cell proliferation in response to (**a**) anti-CD3/anti-CD28 (3 μg/ml) and (**b**) ConA, and B-cell proliferation in response to (**c**) LPS, and (**d**) anti-IgM stimulation. Data are reported as mean stimulation index ratio (SI) ± SEM. Results are based on data from six mice in each group and triplicate measurements for each point

To investigate whether the observed disease phenotype in the congenic mouse strain was accompanied by altered immune cell activation, proliferation was measured in in vitro stimulated splenic B- and T-lymphocytes from naïve and immunized congenic mice. No significant difference in proliferation was observed between spleen cells from the two congenic variant mouse strains upon stimulation with anti-CD3 and anti-CD28 (data not shown). In contrast, splenic lymphocytes from BR.RIIIS/J-*Eae27* mice, immunized with MBP_89–101_ 10 days prior to the experiment, showed increased proliferation compared to the B10.RIII littermate controls, when stimulated in vitro with anti-CD3 and anti-CD28 (*p* = 0.0087) or ConA (*p* = 0.015) (Fig. 3a, b). Similarly, B cell proliferation was increased in immunized BR.RIIIS/J-*Eae27* mice compared to B10.RIII littermates, when stimulated with anti-IgM (*p* = 0.026) and LPS (*p* = 0.019) (Fig. 3c, d). These results demonstrate a difference in the response upon reactivation of lymphocytes from immunized BR.RIIIS/J-*Eae27* and B10.RIII littermates in vitro, and suggest that the *Eae27* region influence lymphocyte activity.

Based on the in vivo and in vitro data showing differences in immune activation between the two *Eae27*-polymorphic mouse strains, bioinformatics search on protein coding genes within the *Eae27* locus, led to selection of genes encoding proteins likely to be involved in immune regulating mechanisms. The *Abl2* gene, located within *Eae27*, encodes the non-receptor tyrosine kinase Arg, which has previously been found highly expressed in a mature B-cell line and, in addition, is important for T-cell function (Bianchi et al., 2002; Gu et al., 2007). Taken together, this led to the hypothesis that *Abl2* is a potential disease candidate gene in *Eae27*.

### Imatinib Has a Therapeutic Effect on EAE

**Fig. 4:**
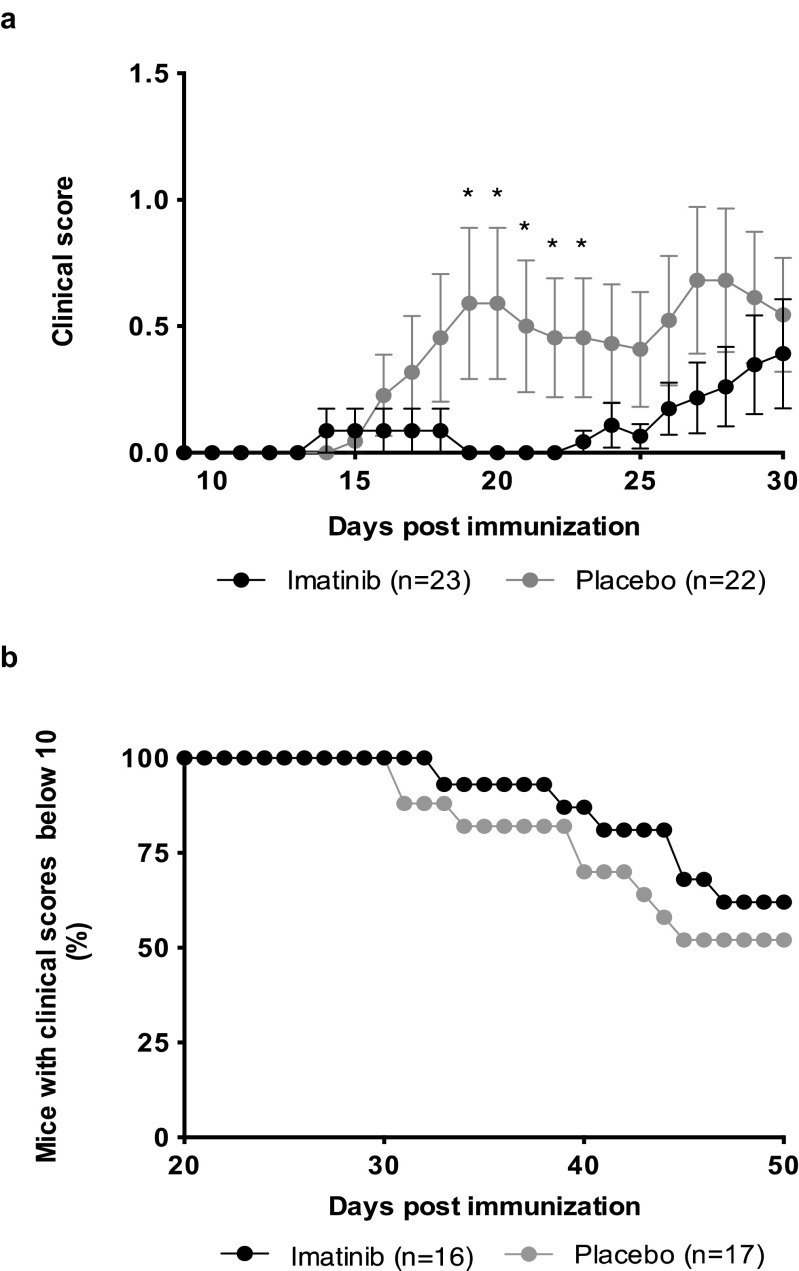
**The effect of imatinib on EAE and CIA development**. Mice received imatinib or placebo 1 day prior to immunization and throughout the study. **a** EAE was induced by immunization with MBP_89–101_ (data represent mean clinical score ± SEM). **b** CIA was induced by immunization with Type II collagen (data represent fraction of mice with a clinical score below 10)

In order to test the hypothesis that the Arg kinase may play a role for the mechanisms causing an autoimmune response observed in EAE, the effect of the Abl kinase inhibitor imatinib was tested in EAE. The group receiving a therapeutic dose of imatinib was protected against EAE in the initial phase of the study compared to the placebo group (*p* = 0.0491) (Fig. 4a). The inhibitor was administered 1 day prior to immunization and throughout the study. Mice receiving the kinase inhibitor had later disease onset (ns) and reduced AUC (*p* = 0,0077), revealing a preventive effect of the drug on EAE. Taken together, the data showed that the selective tyrosine kinase inhibitor imatinib has an ameliorating effect on EAE and indicate a role for Abl kinases in the mechanisms involved in EAE pathogenesis. In contrast to EAE, no significant effect of imatinib on CIA was observed (Fig. 4b).

### Lymphocyte Activation Is Inhibited by Imatinib

**Fig. 5:**
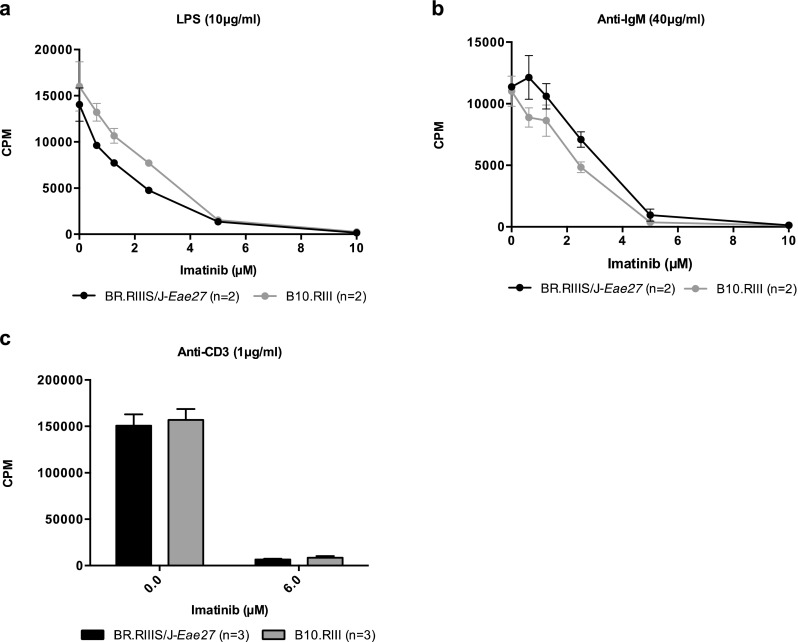
**Inhibition of lymphocyte proliferation with Imatinib**. Imatinib inhibits proliferation of splenic lymphocytes from naïve BR.RIII-*Eae27* and B10.RIII mice upon in vitro stimulation with (**a**) LPS (10 μg/ml), (**b**) anti-IgM (40 μg/ml), or (**c**) anti-CD3/anti-CD28 (1 μg/ml and 3 μg/ml, respectively). Proliferation was measured as ^3^H-thymidine incorporation. Data represent mean CPM ± SEM

To determine whether the effect of the selective tyrosine kinase inhibitor on EAE development was related to cessation of downstream T- and B-cell signaling, the effect of imatinib on immune cell activation was studied in vitro. Proliferation of LPS-, anti-IgM-, or anti-CD3/anti-CD28-stimulated splenic lymphocytes was inhibited by imatinib (Fig. 5a–c). These results demonstrate that Abl kinases are essential actors in the signaling cascades connecting external receptor stimulation to intracellular modifications necessary for establishing an immune response.

### Arg Expression in Brain and Spleen from BR.RIIIS/J-*Eae27* and B10.RIII Mice

**Fig. 6:**
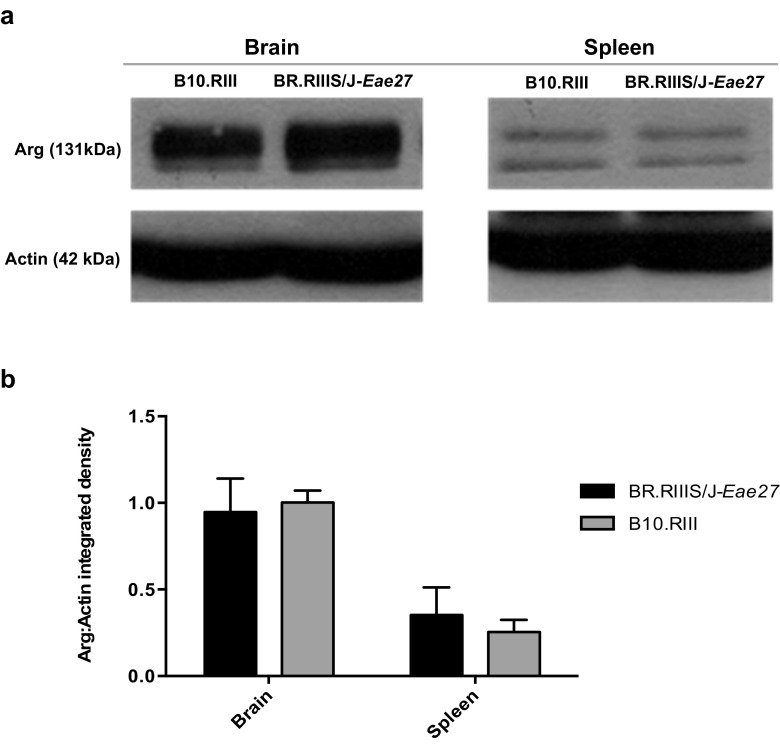
**Arg expression in brain and spleen from BR.RIII-*Eae27*** **and B10.RIII mice**. Western blots showing (**a**) Arg expression in brain and spleen tissue from BR.RIIIS/J-*Eae27* and B10.RIII mice. Molecular mass of Arg and Actin is 131 and 42 kDa, respectively. The two bands observed for Arg are believed to represent splice isoforms (Koleske et al., 1998). **b** The Arg:Actin integrated density is shown graphically. The Arg density is the sum of the two isoform bands

Arg is highly expressed in mouse brain and moderately expressed in spleen (Koleske et al., 1998). In order to test whether the expression of Arg differed between BR.RIIIS/J-*Eae27* and B10.RIII mice and thereby explain the difference in EAE disease phenotype, western blotting was performed on spleen and brain tissue homogenates from the two mouse lines. No difference in Arg protein expression was observed between the *Eae27* genetic variants. The expression of Arg in brain tissue was higher compared to spleen, which correlates with previous findings (Koleske et al., 1998) (Fig. 6a, b).

### A Non-synonymous SNP in the *Abl2* Gene

**Fig. 7:**
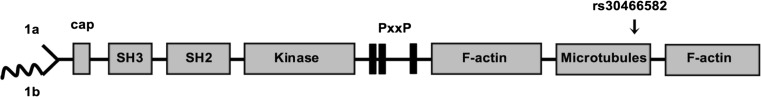
**Domain structure of the Arg protein**. The Arg kinase has two F-actin binding domains, one microtubule-binding domain, and three proline rich (PxxP) motifs in its C-terminal part. The N-terminal part of the protein expresses an SH3, SH2 and kinase domain, forming a domain structure common among tyrosine kinases. The missense SNP, rs30466582, is located within Arg’s microtubule-binding domain close to the C-terminal F-actin-binding domain

Since no differences in expression levels of Arg in brain and spleen were observed in the two congenic variants, a potential role for Arg in EAE susceptibility could therefore be the result of altered protein function. In order to search for polymorphisms between BR.RIIIS/J-*Eae27* and B10.RIII mice, the coding regions of the *Abl2* gene were sequenced. SNP analysis and DNA sequencing confirmed a non-synonymous SNP, rs30466582, in the *Abl2* gene, giving rise to an aa switch in the Arg tyrosine kinase in the B10.RIII strain compared to the RIIIS/J strain. The SNP, rs30466582, is located within a region encoding a microtubules-binding domain, close to an internal F actin-binding domain in the C-terminal part of Arg (Fig. 7). The B10.RIII strain has the amino acid methionine (M) in position 1030, while BR.RIIIS/J-*Eae27* has a valine (V). This aa change could have an influence on the kinase’s capability to reorganize the cytoskeleton.

### The Non-synonymous SNP rs30466582 Does Not Influence Arg Actin Binding Affinity

**Fig. 8:**
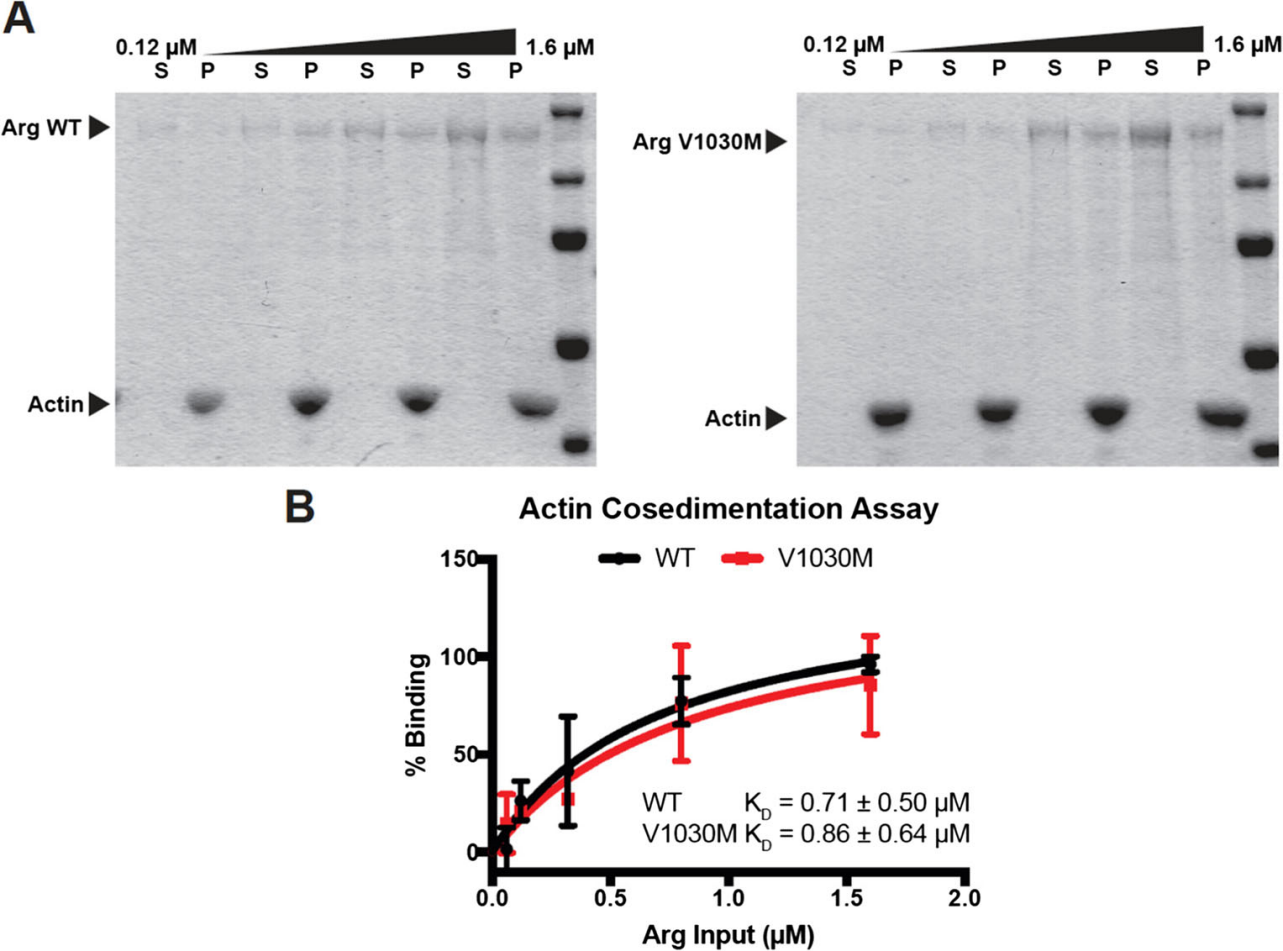
**Arg and Arg V1030 bind with similar affinity to actin filaments**. **a** Representative co-sedimentation assay gels showing increasing amounts of Arg/Arg V1030 M incubated with 1 μM actin. Supernatant (S) and pellet (P) samples for different concentrations are run side by side. **b** Binding of Arg/Arg V1030 to actin in the pellets was quantified from three trials and mean ± SEM is plotted vs. input concentration to calculate the dissociation constant (K_D_). No significant difference was noted in affinity of Arg/Arg V1030 M to actin

We used actin co-sedimentation assays to test whether the Arg V1030 M substitution impacted actin binding. Purified Arg and Arg V1030 M bound with similar affinity to actin filaments (K_D_ = 0.71 ± 0.50 μM for Arg; K_D_ = 0.86 ± 0.64 μM for ArgV1030M) (Fig. 8), and binding saturated at similar ratios of Arg/Arg V1030 M:actin (not shown). These data suggest that the SNP rs30466582, resulting in an aa shift near actin-interacting domains, does not impact actin binding affinity or stoichiometry of binding at saturation.

## Discussion

Abl kinases have been found to play a role for downstream signaling of the T-cell receptor (TCR) by taking part in the phosphorylation cascade initiated upon antigen binding (Zipfel et al., 2004; Gu et al., 2007). Furthermore, c-Abl activity and expression are elevated upon B-cell receptor (BCR) stimulation, and the proliferative response and intracellular calcium flux in splenic B-cells lacking the c-Abl kinase are reduced upon in vitro stimulation with anti-IgM (Matsushita et al., 2008). The role of c-Abl in B-cell signal transduction has been linked to phosphorylation of the BCR co-receptor CD19 (Zipfel et al., 2000; Liberatore and Goff, 2009). c-Abl associates with CD19 through its SH2 domain upon BCR ligation (Zipfel et al., 2000). Considering that c-Abl and Arg share more than 90% structure and domain similarities in their N-terminal parts (Koleske et al., 1998), comprising the SH2, SH3 and kinase domains (Fig. 7), and that the protein level of Arg is high in mature B cells compared to c-Abl (Bianchi et al., 2002), Arg could similarly be an important actor in B-cell activation and proliferation (Andersson and Aksel Jacobsen, 2016). Herein presented data reveal an altered B- and T-cell phenotype in MBP_89–101_ immunized congenic mice, homozygous for *Eae27* inherited from the RIIIS/J donor strain, when stimulated in vitro. The proliferative response to anti-IgM and LPS stimulation in vitro was elevated in splenic B-lymphocytes from MBP_89–101_ immunized BR.RIIIS/J-*Eae27* mice compared to B10.RIII mice. A reduction in proliferation in in vitro LPS- and anti-IgM stimulated B-cells has been documented in CD19-deficient mice (Hasegawa et al., 2001; Yazawa et al., 2003). Whether the reported difference in B-cell proliferation in the *Eae27* congenic mice is related to increased or reduced CD19 signaling in BR.RIIIS/J-*Eae27* and B10.RIII mice upon BCR stimulation, and whether genetic variation within the Arg kinase could influence the signal transduction leading to an altered proliferative response, awaits further investigation.

Inhibition of Abl kinases with imatinib resulted in a dose-dependent inhibition of B- and T-cell proliferation with complete cessation of cell proliferation at the highest concentrations (Fig. 5). These results are consistent with data obtained in other studies (Paniagua et al., 2006; Gu et al., 2007), and confirm that Abl kinases are essential actors in the downstream signaling cascades initiated upon TCR and BCR stimulation. A pharmacological study, testing imatinib in the EAE model, revealed an ameliorating effect of imatinib on disease onset and progression. Obtained results are consistent with data from another study testing the preventive effect of Abl kinase inhibitors on MOG_35–55_-induced EAE (Crespo et al., 2011).

The ability of imatinib to penetrate the blood brain barrier (BBB) is poor in both human and mouse (Dai et al., 2003; le Coutre et al., 2004), suggesting that the observed effect of imatinib on EAE is caused by targeting peripheral lymphocytes before infiltration of the CNS. Interestingly, imatinib has been found to increase BBB integrity. Infiltration of CNS by myelin-specific lymphocytes is a crucial event in MS and EAE pathogenesis, and imatinib enhances BBB integrity in rats induced for EAE (Adzemovic et al., 2013) and experimental subarachnoid hemorrhage (Zhan et al., 2015). The herein presented effect of Abl kinase inhibition on EAE could therefore involve an immune-modulating effect by targeting peripheral lymphocytes, as well as increased BBB integrity. The ameliorating effect of Abl kinase inhibition on EAE development indicates a potential role for inhibition of Abl kinases in future MS treatment.

Interestingly, in the present study the therapeutic effect of imatinib on EAE is declining in the late phase of the study (~day 25 post-immunization). From its widespread use in cancer therapy, development of drug resistance is a known problem in patients treated with imatinib in the clinic (Bixby and Talpaz, 2011). The observed decline in drug effectiveness in the late phase of the EAE study could therefore be a result of beginning drug resistance. Imatinib blocks the enzymatic activity of the Abl kinases by preventing the binding of ATP and inhibition of the catalytic function (Nagar et al., 2002; Lin et al., 2013). Drug resistance to imatinib in human chronic myeloid lymphoma (CML) treatment is to a large extent due to acquired mutations in the *ABL1* gene. Whether the observed resistance to imatinib therapy in the EAE-induced mice followed upon mutations in the *Abl* genes is not known, but additional mechanisms for the declining effect of imatinib treatment might exist. As mentioned above, imatinib has been shown to increase the BBB integrity (Adzemovic et al., 2013). In a study of endothelial barrier function, it was demonstrated that imatinib exerts its beneficial effect on the endothelial barrier by specifically downregulating Arg (Aman et al., 2012). Any mechanism reducing the efficacy of imatinib could, thus, lead to an Arg dependent increase in endothelial barrier dysfunction and cellular influx into the CNS. Another study showed that the number of regulatory T cells is declining upon imatinib treatment (Lu et al., 2017), which could lead to an expansion of autoreactive myelin antigen-specific T-cells overriding the effect of the drug.

Together these results indicate a role for Abl kinases in EAE and strengthen the hypothesis that the *Abl2* gene could be an EAE candidate gene within *Eae27*. In order to test whether the effect of Abl kinase inhibition was due to a general reduction in inflammatory response, the effect of imatinib on CIA was tested. In contrast to previously published data (Paniagua et al., 2006; Terabe et al., 2009), no significant effect on arthritis development was observed in imatinib treated B10.RIII mice. Mice were euthanized when receiving clinical scores above 10 according to predefined humane endpoints. Whether an effect of imatinib on CIA development is observed in the more progressive state of the model could therefore not be determined.

**Fig. 9:**
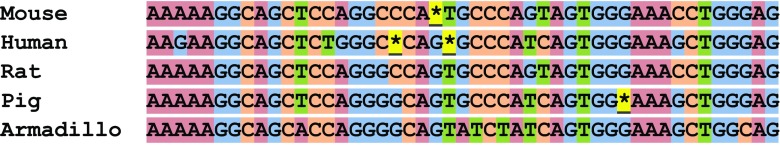
**Alignment of nucleotide sequence 3067–3113 across species**. Nucleotide sequence 3067–3113, flanking the identified missense SNP, rs30466582 (yellow*), in the mouse genome was aligned to human, rat, pig, and armadillo. Two missense SNPs in human are located close to the mouse SNP rs30466582 (ensemble.org)

Sequencing data confirmed a non-synonymous SNP, rs30466582, giving rise to an aa switch from valine to methionine close to the C-terminal F-actin-binding domain of murine Arg. Testing recombinant Arg_1030V and Arg_1030M in an actin co-sedimentation assay, revealed no difference in binding affinity to F-actin between the two recombinant protein variants (Fig. 8). The sequences flanking the identified missense SNP, rs30466582, in mouse are highly conserved in different species. Most mouse strains and other species have a guanine in the position corresponding to aa 1030 (Fig. 9). This results in a less frequent aa at this position in the B10.RIII strain. Interestingly, two missense SNPs have been identified in positions very close to the homologous location of rs30466582 in humans. Whether these missense SNPs would alter the function of Arg has to be elucidated. Interestingly, a genome-wide association study revealed an SNP in the *AXDND1* gene, located less than 150 Mbp from the *ABL2* gene, associated to the age of onset in MS patients (Baranzini et al., 2009). Whether polymorphism(s) in the *ABL2* gene contribute to the association as a result of linkage disequilibrium is still to be investigated.

The results presented in this report reveal an effect of the locus *Eae27* for EAE susceptibility when studied in a congenic mouse strain. In addition to *Abl2*, *Eae27* comprises several genes, and it is not possible to disregard the influence of other genes on EAE development in the *Eae27* congenic mice. The strategy to find candidate genes through genetic studies in an experimental model for MS has, however, resulted in the identification and subsequent studies of one particular candidate gene, the tyrosine kinase Arg, which we believe contributes to the control of lymphocyte activation and CNS inflammation in EAE. This observation should support translational research on inhibition of non-receptor tyrosine kinases, in particular specific inhibition of Arg, in treatment of MS patients. In addition to imatinib, a number of tyrosine kinase inhibitors, approved for cancer treatment and targeting the Abl- and other non-receptor tyrosine kinases, are available and could be potential drug candidates for MS; dasatinib was shown to reduce disease in the initial phase of EAE in mice (Azizi et al., 2015); bosutinib (Golas et al., 2003); and the more Abl specific tyrosine kinase inhibitors nilotinib (Weisberg et al., 2005) and ponatinib (Huang et al., 2010) have not yet been reported for clinical studies in EAE or MS. The present study, together with previous mouse and rat studies, shows that different EAE models are valid for studies of the efficacy of tyrosine kinase inhibitors in neuro-inflammation.

## Conclusion

Our results demonstrate a role for the murine locus *Eae27* in EAE development and lymphocyte activation. We suggest that the *Abl2* gene, encoding the Arg tyrosine kinase, is a disease candidate gene within *Eae27.* This hypothesis is strengthened by the ameliorating effect of an Abl kinase inhibitor on EAE progression using the MBP_89–101_ EAE-inductions protocol in the B10.RIII mouse strain. Furthermore, we confirm the presence of a reported non-synonymous SNP, which results in an aa switch in a protein-binding domain of Arg. We further show, that this aa change has no impact on Arg’s binding affinity for actin. Together, these results show: (−) that studies involving functional genomics in experimental models are important in revealing disease pathways and possible disease therapy, (−) a potential role for Arg in EAE pathogenesis and suggest a possible role for Abl kinase inhibition in future MS treatment.

## Abbreviations

MS: multiple sclerosis
EAE: experimental autoimmune encephalomyelitis
*Arg*: Abelson related gene
MBP: Myelin basic protein
SNP: Single-nucleotide polymorphism
Mbp: Megabase pair
aa: Amino acid
ConA: Concanavalin A
LPS: Lipopolysaccharide

## References

[CR1] Adzemovic MV, Zeitelhofer M, Eriksson U, Olsson T, Nilsson I (2013). Imatinib ameliorates neuroinflammation in a rat model of multiple sclerosis by enhancing blood-brain barrier integrity and by modulating the peripheral immune response. PLoS One.

[CR2] Aman J, van Bezu J, Damanafshan A, Huveneers S, Eringa EC, Vogel SM, Groeneveld J, Vonk Noordegraaf A, van Hinsbergh VW, van Nieuw Amerongen GP (2012). Effective treatment of edema and endothelial barrier dysfunction with imatinib. Circulation.

[CR3] Andersson Å, Aksel Jacobsen F (2016). B-cells and inflammation in the absence of the Abelson related gene (Arg). J Clin Cell Immunol.

[CR4] ANZgene (2009). Genome-wide association study identifies new multiple sclerosis susceptibility loci on chromosomes 12 and 20. Nat Genet.

[CR5] Azizi G, Goudarzvand M, Afraei S, Sedaghat R, Mirshafiey A (2015). Therapeutic effects of dasatinib in mouse model of multiple sclerosis. Immunopharmacol Immunotoxicol.

[CR6] Baranzini SE, Wang J, Gibson RA, Galwey N, Naegelin Y, Barkhof F, Radue EW, Lindberg RLP, Uitdehaag BMG, Johnson MR, Angelakopoulou A, Hall L, Richardson JC, Prinjha RK, Gass A, Geurts JJG, Kragt J, Sombekke M, Vrenken H, Qualley P, Lincoln RR, Gomez R, Caillier SJ, George MF, Mousavi H, Guerrero R, Okuda DT, Cree BAC, Green AJ, Waubant E, Goodin DS, Pelletier D, Matthews PM, Hauser SL, Kappos L, Polman CH, Oksenberg JR (2009). Genome-wide association analysis of susceptibility and clinical phenotype in multiple sclerosis. Hum Mol Genet.

[CR7] Bianchi C, Muradore I, Corizzato M, Cornacchini G, Beretta L, Erba E, Del Monte U, AP R (2002). The expression of the non-receptor tyrosine kinases Arg and c-abl is differently modulated in B lymphoid cells at different stages of differentiation. FEBS Lett.

[CR8] Bixby D, Talpaz M (2011). Seeking the causes and solutions to imatinib-resistance in chronic myeloid leukemia. Leukemia.

[CR9] Bradley WD, Koleske AJ (2009). Regulation of cell migration and morphogenesis by Abl-family kinases: emerging mechanisms and physiological contexts. J Cell Sci.

[CR10] Burkhardt JK, Carrizosa E, Shaffer MH (2008). The actin cytoskeleton in T cell activation. Annu Rev Immunol.

[CR11] Colicelli J (2010). ABL tyrosine kinases: evolution of function, regulation, and specificity. Sci Signal.

[CR12] Crespo O, Kang SC, Daneman R, Lindstrom TM, Ho PP, Sobel RA, Steinman L, Robinson WH (2011). Tyrosine kinase inhibitors ameliorate autoimmune encephalomyelitis in a mouse model of multiple sclerosis. J Clin Immunol.

[CR13] Dai H, Marbach P, Lemaire M, Hayes M, Elmquist WF (2003). Distribution of STI-571 to the brain is limited by P-glycoprotein-mediated efflux. J Pharmacol Exp Ther.

[CR14] Golas JM, Arndt K, Etienne C, Lucas J, Nardin D, Gibbons J, Frost P, Ye F, Boschelli DH, Boschelli F (2003). SKI-606, a 4-anilino-3-quinolinecarbonitrile dual inhibitor of Src and Abl kinases, is a potent antiproliferative agent against chronic myelogenous leukemia cells in culture and causes regression of K562 xenografts in nude mice. Cancer Res.

[CR15] Gu JJ, Zhang N, He YW, Koleske AJ, Pendergast AM (2007). Defective T cell development and function in the absence of Abelson kinases. J Immunol.

[CR16] Harbo HF, Gold R, Tintore M (2013). Sex and gender issues in multiple sclerosis. Ther Adv Neurol Disord.

[CR17] Hasegawa M, Fujimoto M, Poe JC, Steeber DA, Lowell CA, Tedder TF (2001). A CD19-dependent signaling pathway regulates autoimmunity in Lyn-deficient mice. J Immunol.

[CR18] Hernandez SE, Krishnaswami M, Miller AL, Koleske AJ (2004). How do Abl family kinases regulate cell shape and movement?. Trends Cell Biol.

[CR19] Huang Y, Comiskey EO, Dupree RS, Li S, Koleske AJ, Burkhardt JK (2008). The c-Abl tyrosine kinase regulates actin remodeling at the immune synapse. Blood.

[CR20] Huang WS, Metcalf CA, Sundaramoorthi R, Wang Y, Zou D, Thomas RM, Zhu X, Cai L, Wen D, Liu S, Romero J, Qi J, Chen I, Banda G, Lentini SP, Das S, Xu Q, Keats J, Wang F, Wardwell S, Ning Y, Snodgrass JT, Broudy MI, Russian K, Zhou T, Commodore L, Narasimhan NI, Mohemmad QK, Iuliucci J, Rivera VM, Dalgarno DC, Sawyer TK, Clackson T, Shakespeare WC (2010). Discovery of 3-[2-(imidazo[1,2-b]pyridazin-3-yl)ethynyl]-4-methyl-N-{4-[(4-methylpiperazin-1-y l)methyl]-3-(trifluoromethyl)phenyl}benzamide (AP24534), a potent, orally active pan-inhibitor of breakpoint cluster region-abelson (BCR-ABL) kinase including the T315I gatekeeper mutant. J Med Chem.

[CR21] International Multiple Sclerosis Genetics C (2013). Analysis of immune-related loci identifies 48 new susceptibility variants for multiple sclerosis. Nat Genet.

[CR22] Hafler DA, Compston A, Sawcer S, Lander ES, Daly MJ, De Jager PL, de Bakker PI, Gabriel SB, Mirel DB, Ivinson AJ, Pericak-Vance MA, Gregory SG, Rioux JD, JL MC, Haines JL, Barcellos LF, Cree B, Oksenberg JR, Hauser SL, International Multiple Sclerosis Genetics C (2007). Risk alleles for multiple sclerosis identified by a genomewide study. N Engl J Med.

[CR23] Jansson L, Olsson T, Hojeberg B, Holmdahl R (1991). Chronic experimental autoimmune encephalomyelitis induced by the 89-101 myelin basic protein peptide in B10RIII (H-2r) mice. Eur J Immunol.

[CR24] Karlsson J, Zhao X, Lonskaya I, Neptin M, Holmdahl R, Andersson A (2003). Novel quantitative trait loci controlling development of experimental autoimmune encephalomyelitis and proportion of lymphocyte subpopulations. J Immunol.

[CR25] Koleske AJ, Gifford AM, Scott ML, Nee M, Bronson RT, Miczek KA, Baltimore D (1998). Essential roles for the Abl and Arg tyrosine kinases in neurulation. Neuron.

[CR26] Kruh GD, King CR, Kraus MH, Popescu NC, Amsbaugh SC, McBride WO, Aaronson SA (1986). A novel human gene closely related to the abl proto-oncogene. Science.

[CR27] le Coutre P, Kreuzer KA, Pursche S, Bonin M, Leopold T, Baskaynak G, Dorken B, Ehninger G, Ottmann O, Jenke A, Bornhauser M, Schleyer E (2004). Pharmacokinetics and cellular uptake of imatinib and its main metabolite CGP74588. Cancer Chemother Pharmacol.

[CR28] Liberatore RA, Goff SP (2009). C-Abl-deficient mice exhibit reduced numbers of peritoneal B-1 cells and defects in BCR-induced B cell activation. Int Immunol.

[CR29] Lin YL, Meng Y, Jiang W, Roux B (2013). Explaining why Gleevec is a specific and potent inhibitor of Abl kinase. Proc Natl Acad Sci U S A.

[CR30] Lindvall T, Nandakumar KS, Yousefi K, Holmdahl R, Andersson A (2011). An encephalomyelitis-specific locus on chromosome 16 in mouse controls disease development and expression of immune-regulatory genes. J Neuroimmunol.

[CR31] Lu Z, Xu N, Zhou X, Gao G, Li L, Huang J, Li Y, Lu Q, He B, Pan C, Liu X (2017). Therapeutic immune monitoring of CD4(+)CD25(+) T cells in chronic myeloid leukemia patients treated with tyrosine kinase inhibitors. Oncol Lett.

[CR32] Markel P, Shu P, Ebeling C, Carlson GA, Nagle DL, Smutko JS, Moore KJ (1997). Theoretical and empirical issues for marker-assisted breeding of congenic mouse strains. Nat Genet.

[CR33] Matsushita T, Yanaba K, Bouaziz JD, Fujimoto M, Tedder TF (2008). Regulatory B cells inhibit EAE initiation in mice while other B cells promote disease progression. J Clin Invest.

[CR34] Miller AL, Wang Y, Mooseker MS, Koleske AJ (2004). The Abl-related gene (Arg) requires its F-actin-microtubule cross-linking activity to regulate lamellipodial dynamics during fibroblast adhesion. J Cell Biol.

[CR35] Miller MM, Lapetina S, MacGrath SM, Sfakianos MK, Pollard TD, Koleske AJ (2010). Regulation of actin polymerization and adhesion-dependent cell edge protrusion by the Abl-related gene (Arg) tyrosine kinase and N-WASp. Biochemistry.

[CR36] Nagar B, Bornmann WG, Pellicena P, Schindler T, Veach DR, Miller WT, Clarkson B, Kuriyan J (2002). Crystal structures of the kinase domain of c-Abl in complex with the small molecule inhibitors PD173955 and imatinib (STI-571). Cancer Res.

[CR37] Paniagua RT, Sharpe O, Ho PP, Chan SM, Chang A, Higgins JP, Tomooka BH, Thomas FM, Song JJ, Goodman SB, Lee DM, Genovese MC, Utz PJ, Steinman L, Robinson WH (2006). Selective tyrosine kinase inhibition by imatinib mesylate for the treatment of autoimmune arthritis. JClinInvest.

[CR38] Patsopoulos NA, Barcellos LF, Hintzen RQ, Schaefer C, van Duijn CM, Noble JA, Raj T, Gourraud PA, Stranger BE, Oksenberg J, Olsson T, Taylor BV, Sawcer S, Hafler DA, Carrington M, De Jager PL, de Bakker PI, Imsgc, Anzgene (2013). Fine-mapping the genetic association of the major histocompatibility complex in multiple sclerosis: HLA and non-HLA effects. PLoS Genet.

[CR39] Rioux JD (2009). Mapping of multiple susceptibility variants within the MHC region for 7 immune-mediated diseases. Proc Natl Acad Sci U S A.

[CR40] Rogner UC, Avner P (2003). Congenic mice: cutting tools for complex immune disorders. Nat Rev Immunol.

[CR41] Spudich JA, Watt S (1971). The regulation of rabbit skeletal muscle contraction. I. Biochemical studies of the interaction of the tropomyosin-troponin complex with actin and the proteolytic fragments of myosin. J Biol Chem.

[CR42] Sundvall M, Jirholt J, Yang HT, Jansson L, Engstrom A, Pettersson U, Holmdahl R (1995). Identification of murine loci associated with susceptibility to chronic experimental autoimmune encephalomyelitis. Nat Genet.

[CR43] Terabe F, Kitano M, Kawai M, Kuwahara Y, Hirano T, Arimitsu J, Hagihara K, Shima Y, Narazaki M, Tanaka T, Kawase I, Sano H, Ogata A (2009). Imatinib mesylate inhibited rat adjuvant arthritis and PDGF-dependent growth of synovial fibroblast via interference with the Akt signaling pathway. Mod Rheumatol.

[CR44] Wakeland E, Morel L, Achey K, Yui M, Longmate J (1997). Speed congenics: a classic technique in the fast lane (relatively speaking). Immunol Today.

[CR45] Wang Y, Miller AL, Mooseker MS, Koleske AJ (2001). The Abl-related gene (Arg) nonreceptor tyrosine kinase uses two F-actin-binding domains to bundle F-actin. Proc Natl Acad Sci U S A.

[CR46] Wang Q, Zimmerman EI, Toutchkine A, Martin TD, Graves LM, Lawrence DS (2010). Multicolor monitoring of dysregulated protein kinases in chronic myelogenous leukemia. ACS Chem Biol.

[CR47] Weisberg E, Manley PW, Breitenstein W, Brüggen J, Cowan-Jacob SW, Ray A, Huntly B, Fabbro D, Fendrich G, Hall-Meyers E, Kung AL, Mestan J, Daley GQ, Callahan L, Catley L, Cavazza C, Mohammed A, Neuberg D, Wright RD, Gilliland DG, Griffin JD (2005). Characterization of AMN107, a selective inhibitor of native and mutant Bcr-Abl. Cancer Cell.

[CR48] Yazawa N, Fujimoto M, Sato S, Miyake K, Asano N, Nagai Y, Takeuchi O, Takeda K, Okochi H, Akira S, Tedder TF, Tamaki K (2003). CD19 regulates innate immunity by the toll-like receptor RP105 signaling in B lymphocytes. Blood.

[CR49] Zhan Y, Krafft PR, Lekic T, Ma Q, Souvenir R, Zhang JH, Tang J (2015). Imatinib preserves blood-brain barrier integrity following experimental subarachnoid hemorrhage in rats. J Neurosci Res.

[CR50] Zipfel PA, Grove M, Blackburn K, Fujimoto M, Tedder TF, Pendergast AM (2000). The c-Abl tyrosine kinase is regulated downstream of the B cell antigen receptor and interacts with CD19. J Immunol.

[CR51] Zipfel PA, Zhang W, Quiroz M, Pendergast AM (2004). Requirement for Abl kinases in T cell receptor signaling. Curr Biol.

